# Revealing the relationship between photoelectrochemical performance and interface hole trapping in CuBi_2_O_4_ heterojunction photoelectrodes†

**DOI:** 10.1039/d0sc03030a

**Published:** 2020-09-14

**Authors:** Angang Song, Igal Levine, Roel van de Krol, Thomas Dittrich, Sean P. Berglund

**Affiliations:** Institute for Solar Fuels, Helmholtz-Zentrum Berlin für Materialien und Energie GmbH Hahn-Meitner-Platz 1 14109 Berlin Germany sean.berglund@gmail.com; Institut für Chemie, Technische Universität Berlin Straße des 17. Juni 124 10623 Berlin Germany; Institute for Silicon Photovoltaics, Helmholtz-Zentrum Berlin für Materialien und Energie GmbH Kekuléstr. 5 12489 Berlin Germany dittrich@helmholtz-berlin.de

## Abstract

p-Type CuBi_2_O_4_ is considered a promising metal oxide semiconductor for large-scale, economic solar water splitting due to the optimal band structure and low-cost fabrication. The main challenge in utilizing CuBi_2_O_4_ as a photoelectrode for water splitting, is that it must be protected from photo-corrosion in aqueous solutions, an inherent problem for Cu-based metal oxide photoelectrodes. In this work, several buffer layers (CdS, BiVO_4_, and Ga_2_O_3_) were tested between CuBi_2_O_4_ and conformal TiO_2_ as the protection layer. RuO_*x*_ was used as the co-catalyst for hydrogen evolution. Factors that limit the photoelectrochemical performance of the CuBi_2_O_4_/TiO_2_/RuO_*x*_, CuBi_2_O_4_/CdS/TiO_2_/RuO_*x*_, CuBi_2_O_4_/BiVO_4_/TiO_2_/RuO_*x*_ and CuBi_2_O_4_/Ga_2_O_3_/TiO_2_/RuO_*x*_ heterojunction photoelectrodes were revealed by comparing photocurrents, band offsets, and directed charge transfer measured by modulated surface photovoltage spectroscopy. For CuBi_2_O_4_/Ga_2_O_3_/TiO_2_/RuO_*x*_ photoelectrodes, barriers for charge transfer strongly limited the performance. In CuBi_2_O_4_/CdS/TiO_2_/RuO_*x*_, the absence of hole traps resulted in a relatively high photocurrent density and faradaic efficiency for hydrogen evolution despite the presence of pronounced deep defect states at the CuBi_2_O_4_/CdS interface. Hole trapping limited the performance moderately in CuBi_2_O_4_/BiVO_4_/TiO_2_/RuO_*x*_ and strongly in CuBi_2_O_4_/TiO_2_/RuO_*x*_ photoelectrodes. For the first time, our results show that hole trapping is a key factor that must be addressed to optimize the performance of CuBi_2_O_4_-based heterojunction photoelectrodes.

## Abstract 2

CdS, BiVO_4_, and Ga_2_O_3_ buffer layers were tested between CuBi_2_O_4_ and TiO_2_ in heterojunction photoelectrodes. Photoelectrochemical analysis and modulated surface photovoltage spectroscopy revealed that interface hole traps impacted device performance.

## Graphical abstract

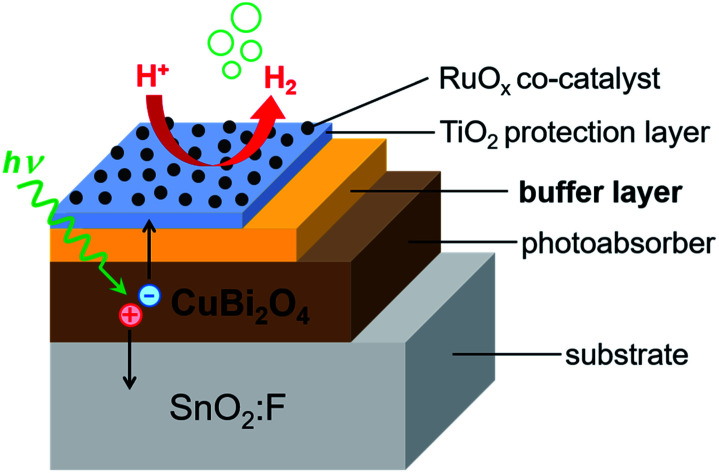

## Introduction

Copper bismuth oxide (CuBi_2_O_4_) is a promising photoabsorber for photoelectrochemical (PEC) water splitting due to its optimal optical bandgap (1.5–1.8 eV), positive photocurrent onset potential (more positive than 1 V *vs.* RHE), and Earth-abundant chemical composition.^1–4^ However, several limitations in CuBi_2_O_4_ must be overcome to improve its performance as a photocathode for the hydrogen evolution reaction. Perhaps the greatest limitation of CuBi_2_O_4_ is its susceptibility to photo-corrode under illumination in aqueous solutions, which is a common problem for Cu-based metal oxide photoelectrodes.^5–8^ Surface modification *via* the formation of a heterojunction with a suitable buffer layer and/or an n-type protection layer could be highly effective at overcoming this limitation of CuBi_2_O_4_ based on previous reports on other Cu-based metal oxide photocathodes such as Cu_2_O, CuO and CuFeO_4_ as well as Si-based photoelectrodes.^9–13^

To be effective, a suitable buffer layer and/or protective layer must cover CuBi_2_O_4_ conformally without any pinholes and have energy band positions that match favorably to allow for efficient transport of charge carriers across the solid-state and semiconductor–electrolyte interfaces. TiO_2_ deposited by atomic layer deposition (ALD) has been reported as an excellent protective layer for unstable photoelectrodes while simultaneously allowing for efficient electron transfer to the electrolyte under the PEC conditions for hydrogen evolution.^11,14–16^ Studies have shown that TiO_2_-protected Cu_2_O-based photocathodes exhibit a relatively high photocurrent density and significantly enhanced stability when using various buffer layers between Cu_2_O and TiO_2_ such as ZnO and Al doped ZnO (AZO).^15,17,18^ However, relatively poor photocurrent onset potentials (0.45–0.55 V *vs.* RHE) were obtained in this structure due to the small photovoltage produced by the heterojunctions. It has been shown that the use of ZnS as a buffer layer between Cu_2_O and TiO_2_ can increase the photovoltage at the multilayer/electrolyte junction thereby shifting the onset potential cathodically to 0.7 V *vs.* RHE.^19^ The introduction of Ga_2_O_3_ as a buffer layer between Cu_2_O and TiO_2_ can improve the photovoltage even further (open-circuit voltage up to 1.2 V for Cu_2_O solar cells^20^ and photocurrent onset above 1.0 V *vs.* RHE for photocathodes).^21–23^ In another work for CuO-based heterojunction photocathodes, ZnO showed rather poor performance as a buffer layer, in contrast to CdS.^24^

The band positions of the various layers within TiO_2_-protected heterojunction photocathodes are crucial in determining the overall performance. The photovoltage of the device is ultimately limited by the difference in the Fermi level of the photoabsorber and the conduction band of either the buffer layer or the TiO_2_ protective layer, depending on which has a lower conduction band.^5^ Furthermore, numerous studies have emphasized the importance of band alignment between the photoabsorber and the buffer layer. In addition to band alignment, other key factors related to recombination and/or trapping at the layer interfaces can contribute to the overall performance. For example, it was recently shown by model calculations that Al-doped ZnO (AZO) buffer layers between Cu_2_O and TiO_2_ should enable a higher photovoltage than Ga_2_O_3_ buffer layers, but AZO may induce an interface recombination layer that hinders charge transfer and thus decreases the photovoltage.^9^ Therefore, in addition to optimal band energy alignment, heterojunction interface layers must be high-quality and possess low interfacial trap densities to maximize device performance. Incidentally, compared to band positions, defect states and recombination sites at interfaces are often more difficult to characterize. Modulated surface photovoltage (SPV) spectroscopy can be used to provide information about electronic transitions from which photogeneration can take place and about the direction of charge separation even over very short distances and with very high sensitivity.^25,26^

**Fig. 1:**
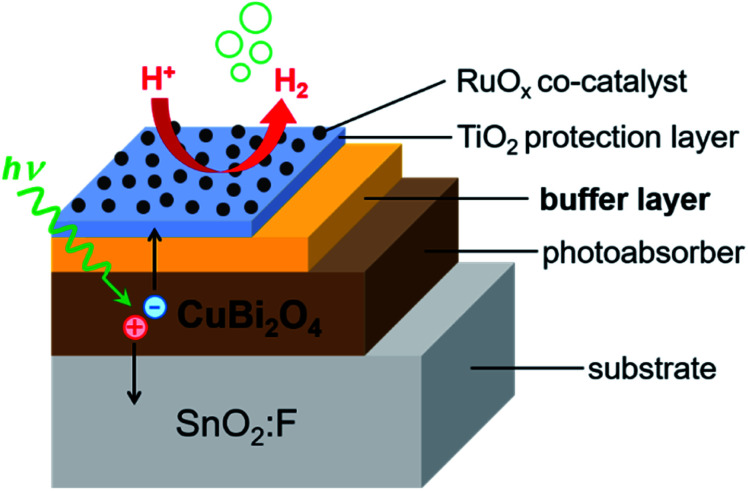
Schematic of the layer stacking for the heterojunction photoelectrodes analyzed in this work. A TiO_2_ protection layer was used in all cases, which was deposited either directly on the CuBi_2_O_4_ absorber, or on the Ga_2_O_3_, BiVO_4_, or CdS buffer layer. RuO_*x*_ was used as a co-catalyst for the hydrogen evolution reaction. The substrate was SnO_2_ : F, fluorine-doped tin oxide (FTO).

In order to obtain information about dominant limiting factors in heterojunction photoelectrodes with CuBi_2_O_4_ as the absorber layer and ALD-deposited TiO_2_ as the protective layer, we compared how different buffer layers (CdS, BiVO_4_, and Ga_2_O_3_) affected the photocurrent density (with RuO_*x*_ as a co-catalyst layer for H^+^ reduction reaction) as well as the behavior of modulated SPV spectra. Fig. 1 shows the layer stacking of the various heterojunction photoelectrodes that were tested in this work. The energy positions of the valence band edges in the separate layers were measured by photoelectron spectroscopy. Our results show that, in addition to suitable band alignment for charge transfer, preferential trapping of holes at CuBi_2_O_4_/TiO_2_, CuBi_2_O_4_/BiVO_4_ and CuBi_2_O_4_/Ga_2_O_3_ interfaces drastically limits the photocurrent of the corresponding PEC systems. In contrast, preferential trapping of electrons at the CuBi_2_O_4_/CdS interface limits the photocurrent to a much lesser extent despite the appearance of pronounced deep defect states.

## Results and discussion

### Photoelectrochemical analysis

In this work we performed PEC measurements on bare CuBi_2_O_4_ photocathodes in addition to the CuBi_2_O_4_/CdS/TiO_2_/RuO_*x*_, CuBi_2_O_4_/Ga_2_O_3_/TiO_2_/RuO_*x*_, CuBi_2_O_4_/BiVO_4_/TiO_2_/RuO_*x*_ and CuBi_2_O_4_/TiO_2_/RuO_*x*_ heterojunction photoelectrodes. They were measured under chopped AM 1.5 illumination in 0.3 M K_2_SO_4_ and 0.2 M phosphate buffer (pH 6.8) with Ar bubbling to purge dissolved oxygen from the electrolyte. As shown in Fig. S1a in the ESI,† the bare CuBi_2_O_4_ electrode exhibited a relatively large cathodic photocurrent density of −1 mA cm^−2^ at 0.4 V *vs.* RHE under visible light illumination for the chopped linear sweep voltammetry (LSV) measurement. However, the photocurrent density decayed rapidly, as shown in the constant potential measurement under illumination at 0.6 V *vs.* RHE (see Fig. S1b†). Based on previous reports this is attributed to the reduction of Cu^2+^ to Cu^1+^ and/or Cu in aqueous solution.^1,27–29^ After 30 minutes the photocurrent density was only 6.4% of the initial value and by the end of the 5 hour measurement the illuminated area of the photoelectrode was transparent (see the inset of Fig. S1b†) presumably because the reduced copper dissolved into the electrolyte. Differential mass spectrometry (DEMS) measurements confirmed that the bare CuBi_2_O_4_ photocathodes did not produce a detectable amount of hydrogen (see Fig. S1c†).

**Fig. 2:**
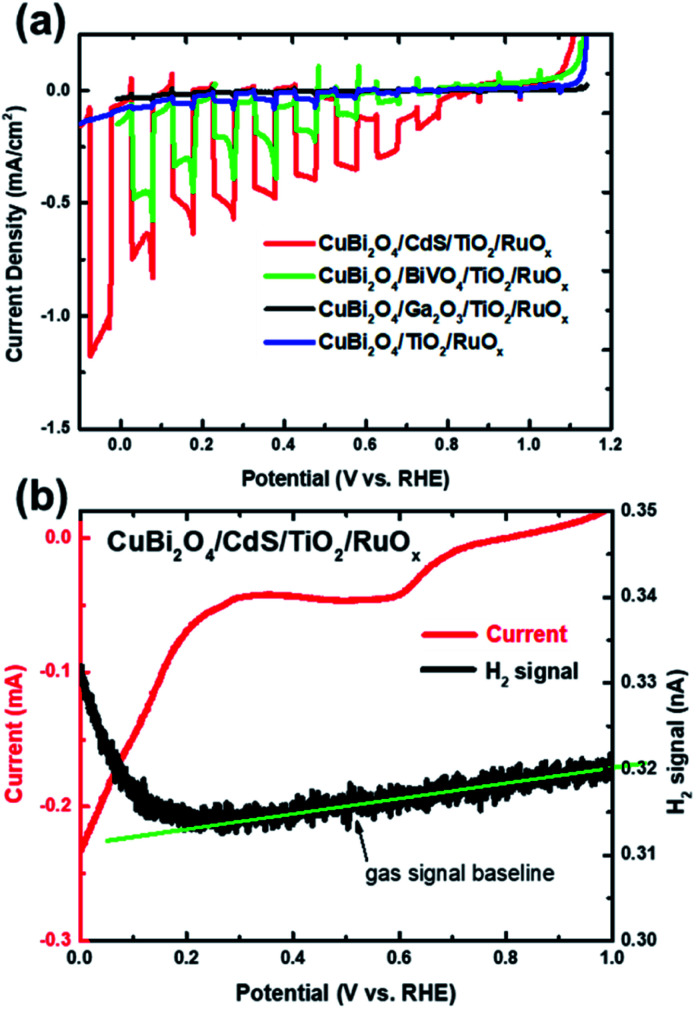
(a) Chopped LSV scans for a CuBi_2_O_4_/CdS/TiO_2_/RuO_*x*_ (red), CuBi_2_O_4_/BiVO_4_/TiO_2_/RuO_*x*_ (green), CuBi_2_O_4_/Ga_2_O_3_/TiO_2_/RuO_*x*_ (black) and CuBi_2_O_4_/TiO_2_/RuO_*x*_ photocathode (blue) under simulated AM1.5 illumination (these are representative measurements of at least 4 samples for each condition). (b) Differential electrochemical mass spectrometry (DEMS) LSV scans for a CuBi_2_O_4_/CdS/TiO_2_/RuO_*x*_ photocathode with illumination by a xenon lamp, showing current (red) and H_2_ signal (black). Measurements were performed in three-electrode configuration in 0.3 M K_2_SO_4_ and 0.2 M phosphate buffer electrolyte (pH 6.8) with Ar bubbling.

The chopped LSV curves for the CuBi_2_O_4_/CdS/TiO_2_/RuO_*x*_, CuBi_2_O_4_/Ga_2_O_3_/TiO_2_/RuO_*x*_, CuBi_2_O_4_/BiVO_4_/TiO_2_/RuO_*x*_ and CuBi_2_O_4_/TiO_2_/RuO_*x*_ heterojunction photoelectrodes are shown in Fig. 2a. Unlike the bare CuBi_2_O_4_ photocathode, the heterojunction photoelectrodes showed minimal dark currents at potentials more negative than 0.35 V *vs.* RHE, indicating that the ALD-deposited TiO_2_ layer effectively inhibits the electrochemical corrosion of the underlying CuBi_2_O_4_. However, the photocurrent generated from the CuBi_2_O_4_/TiO_2_/RuO_*x*_ and CuBi_2_O_4_/Ga_2_O_3_/TiO_2_/RuO_*x*_ photocathodes (blue and black lines) were very small (*ca.* −0.04 mA cm^−2^ and −0.01 mA cm^−2^ at 0 V *vs.* RHE, respectively). This indicates that the CuBi_2_O_4_/TiO_2_ and the CuBi_2_O_4_/Ga_2_O_3_ or Ga_2_O_3_/TiO_2_ interfaces in these samples do not effectively charge transport to the RuO_*x*_ co-catalyst layer and into the electrolyte. The CuBi_2_O_4_/BiVO_4_/TiO_2_/RuO_*x*_ photoelectrode (green line) shows significantly higher photocurrent density (*ca.* −0.5 mA cm^−2^ at 0 V *vs.* RHE) while the CuBi_2_O_4_/CdS/TiO_2_/RuO_*x*_ photoelectrode (red line) shows by far the highest photoactivity with a plateau in photocurrent density at *ca.* −1 mA cm^2^ at 0 V *vs.* RHE. It also has the most positive photocurrent onset at 0.8 V *vs.* RHE. For the CuBi_2_O_4_/CdS/TiO_2_/RuO_*x*_ photoelectrode the bump in the dark current around 0.5–0.6 V *vs.* RHE is likely due to the reduction of dissolved oxygen that could not be completely purged from the PEC cell by Ar bubbling,^5^ or another reduction process on the electrode surface such as the pre-reduction of the RuO_*x*_ catalyst.^16^ The DEMS measurement (Fig. 2b) shows that when the photocurrent increases cathodically at potentials negative of 0.3 V *vs.* RHE (red line) there is a simultaneous increase in H_2_ signal (black line) confirming that the CuBi_2_O_4_/CdS/TiO_2_/RuO_*x*_ heterojunction photoelectrode can evolve hydrogen photoelectrochemically. The faradaic efficiency was estimated to be 92% based on calibration of the DEMS using a Pt metal electrode.

Note that the photocurrent density directly before the onset of dark current from electrochemical proton reduction (*ca.* −1 mA cm^2^ at 0 V *vs.* RHE in Fig. 2a) is close to the maximum photocurrent density for bare CuBi_2_O_4_ before the onset of dark current from electrochemical corrosion (*ca.* −1 mA cm^−2^ at 0.4 V *vs.* RHE in Fig. S1a†). This implies that most of the electrons that are photogenerated in CuBi_2_O_4_, which previously participated in the corrosion reaction (Cu^2+^ → Cu^1+^ and/or Cu), are successfully injected into the CdS buffer and TiO_2_ protection layers, and eventually into the electrolyte to drive the hydrogen evolution reaction.

In order to investigate the long-term stability of the CuBi_2_O_4_/CdS/TiO_2_/RuO_*x*_ photocathodes, the photocurrent density was measured at a constant potential of 0.0 V *vs.* RHE for 5 hours with intermittent chopping of the front illumination (see Fig. S2 in the ESI†). The measurement shows a decay in photocurrent which was more than 100 times slower than for the bare CuBi_2_O_4_ photocathode (Fig. S1b†). A significant part of the decay was attributed to deterioration of the RuO_*x*_ co-catalyst as demonstrated by partial restoration of the photocurrent after re-deposition of RuO_*x*_ after 3 hours. At the end of the 5 hour measurement, and after the single re-deposition of the RuO_*x*_ co-catalyst, 30% of the initial photocurrent density was preserved. Visually the illuminated area of the CuBi_2_O_4_/CdS/TiO_2_/RuO_*x*_ photoelectrode was almost indistinguishable from the non-illuminated area (see the inset of Fig. S2†). Under scanning electron microscope (SEM) there were no significant morphological differences between areas of the sample that were and were not PEC tested other than a slight smoothing of the electrode surface after PEC testing (see Fig. S3†). Therefore, the ALD-deposited TiO_2_ layer effectively blocked the contact between CuBi_2_O_4_ and the aqueous electrolyte and hindered photo-corrosion. This was also the case for the other layer systems. For comparison, photos and SEM images of the CuBi_2_O_4_/CdS/TiO_2_, CuBi_2_O_4_/Ga_2_O_3_/TiO_2_ and CuBi_2_O_4_/BiVO_4_/TiO_2_ photoelectrodes are shown in Fig. S4–S6,† respectively.

### Band alignment

In order to estimate the band positions of the different materials in the heterojunction photoelectrodes, the energy band offsets were measured through a combination of X-ray photoelectron spectroscopy (XPS), ultraviolet photoelectron spectroscopy (UPS), UV-visible spectrophotometry, and Mott–Schottky analysis (including work from previous studies). Fig. S7a† shows the XPS spectrum for Cu at the surface of bare CuBi_2_O_4_. There are two main peaks at 933.8 and 953.6 eV corresponding to the Cu 2p_3/2_ and 2p_1/2_ levels, both of which were attributed to the presence of the Cu^2+^ state on the surface.^30^ The binding energies of Cd 3d, Ga 3d, V 2p and Ti 2p core levels of CdS, Ga_2_O_3_, BiVO_4_ and TiO_2_ are measured to be 406.3, 21.2, 516.7 and 459.3 eV, respectively (see Fig. S7b–e†). Fig. S8† shows the XPS survey results for the stack samples of the CuBi_2_O_4_/CdS/TiO_2_, CuBi_2_O_4_/Ga_2_O_3_/TiO_2_, CuBi_2_O_4_/BiVO_4_/TiO_2_ and CuBi_2_O_4_/TiO_2_. The presence of strong Ti peaks and the absence of Cu, Cd, Ga and V peaks in the survey spectra indicates that the ALD-deposited TiO_2_ layer fully covers the buffer layers.

For the band positions of bare CuBi_2_O_4_, we used our previously reported values from Mott–Schottky analysis and UPS measurements, to estimate a conduction band (*E*_C_) of −0.3 V *vs.* RHE and a valence band (*E*_V_) of 1.2 V *vs.* RHE.^4,5,31^ The flat-band potential (*φ*_fb_) of our spray-deposited BiVO_4_ films is 0.37 V *vs.* RHE,^5,32^ which can then be used to estimate the Fermi level (*E*_F_) while taking into account the potential drop across the Helmholtz layer.^5,33^ Fig. S9a–c† show the UPS spectra of CdS, Ga_2_O_3_ and TiO_2_ thin films on FTO substrate measured with a 2 V bias. The work function (*Φ*), defined as the difference between the vacuum energy level and Fermi level (*E*_F_), can be derived from the low kinetic energy cut-off in the secondary emission feature. The photon energy of the UV source (He I discharge) was 21.21 eV. Given that the Fermi level at the surface of these overlayers is considered independently of the spectrometer, the work function is determined to be 21.21 – 2 – secondary emission cut-off (SEC). The work function of CdS, Ga_2_O_3_ and TiO_2_ thin films are calculated to be 4.12, 3.63 and 4.57 eV, respectively. Using 4.5 eV *vs.* vacuum as the reference value for the electrochemical proton reduction (0.0 V *vs.* RHE) we can estimate the Fermi energies of the CdS, Ga_2_O_3_ and TiO_2_ layers at approximately −0.38, −0.87 and 0.07 V *vs.* RHE, respectively. These values are in agreement with the literature.^34–36^

The valence band positions with respect to the position of the Fermi level, *E*_F_ − *E*_V_, were determined by linear extrapolation of the UPS spectrum at the low binding energy side to the binding energy axis (see Fig. S10†). *E*_F_ − *E*_V_ for CdS, BiVO_4_, Ga_2_O_3_ and TiO_2_ thin films are calculated to be 2.5, 2.4, 4.6, and 3.3 eV, respectively, which is consistent with previously reported values.^37,38^ Since CdS has a bandgap of 2.4–2.5 eV this would place the conduction band very close to the Fermi level at −0.38 V *vs.* RHE, which is within the wide range of previously reported *E*_C_ values for CdS.^5,39,40^ The experimentally determined band energy values for all samples are summarized in Table S1 in the ESI† along with values from the literature.

**Fig. 3:**
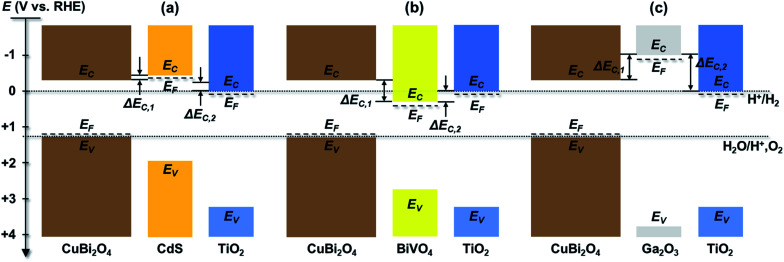
Estimated band diagrams for systems containing (a) CuBi_2_O_4_/CdS/TiO_2_, (b) CuBi_2_O_4_/BiVO_4_/TiO_2_ and (c) CuBi_2_O_4_/Ga_2_O_3_/TiO_2_ in relation to electrochemical redox potentials for proton reduction (H^+^/H_2_) and water oxidation (H^+^, O_2_/H_2_O). *E*_F_ is the Fermi level, *E*_C_ is the conduction band, *E*_V_ is the valence band, and Δ*E*_C,1_ and Δ*E*_C,2_ are the conduction band offsets.

Using the values given in Table S1,† the energies of conduction and valence bands were obtained and illustrated in Fig. 3 for the individual layers in the CuBi_2_O_4_/CdS/TiO_2_, CuBi_2_O_4_/Ga_2_O_3_/TiO_2_ and CuBi_2_O_4_/BiVO_4_/TiO_2_ heterojunction photoelectrodes in relation to the electrochemical redox potentials for proton reduction (H^+^/H_2_) and water oxidation (H^+^, O_2_/H_2_O) at 0.0 and 1.23 V *vs.* RHE, respectively. Since the conduction band of CuBi_2_O_4_ is at −0.3 V *vs.* RHE and the Fermi level of TiO_2_ is at approximately 0.0 V *vs.* RHE, all of the heterojunctions shown in Fig. 3 should be thermodynamically capable of reducing H^+^ as long as photogenerated electrons can be transported to the TiO_2_ surface. However, there are differences in the conduction band offsets between CuBi_2_O_4_ and each buffer layer (Δ*E*_C,1_) and between TiO_2_ and each buffer layer (Δ*E*_C,2_). For a wide range of heterojunctions, including CuO/TiO_2_ junctions, it has been shown that a high conduction band offset can promote high interface recombination and therefore inhibit charge transport.^41^ Better band alignment of the CuBi_2_O_4_/buffer layer provides a larger driving force for charge transport in CuBi_2_O_4_, results in a smaller concentration of holes near the interface and reduces the interfacial recombination. For all of the systems shown in Fig. 3, Δ*E*_C,1_ is by far the smallest between CuBi_2_O_4_ and CdS (less than 0.2 eV) while it is approximately 0.57 eV between CuBi_2_O_4_ and BiVO_4_ and –0.67 eV between CuBi_2_O_4_ and Ga_2_O_3_ assuming that *E*_C_ is approximately 0.1 eV above *E*_F_ in the buffer layers. The significantly higher conduction band of Ga_2_O_3_ compared to CuBi_2_O_4_ creates an energy barrier, thus significantly limiting the charge separation efficiency and the injection of the photogenerated electrons to the TiO_2_, which increases the recombination rate of photogenerated electrons and holes at the CuBi_2_O_4_/Ga_2_O_3_/TiO_2_ interfaces, especially if they contain defect states. This alone is enough to explain the poor performance of the CuBi_2_O_4_/Ga_2_O_3_/TiO_2_/RuO_*x*_ photoelectrode compared to the others. For all of the heterojunctions shown in Fig. 3, Δ*E*_C,2_ is also smallest with CdS as the buffer layer. For the CuBi_2_O_4_/BiVO_4_/TiO_2_ system, transport of photogenerated electrons from CuBi_2_O_4_ to BiVO_4_ is thermodynamically favorable, but the Δ*E*_C,2_ value of −0.27 eV may hinder transport of these electrons into TiO_2_. Overall, the relatively small conduction band offsets between CuBi_2_O_4_, CdS, and TiO_2_ makes the CuBi_2_O_4_/CdS/TiO_2_ heterojunction photoelectrode more favorable for obtaining a high photovoltage and transporting charges compared to the CuBi_2_O_4_/Ga_2_O_3_/TiO_2_/RuO_*x*_ and CuBi_2_O_4_/BiVO_4_/TiO_2_/RuO_*x*_ photoelectrodes.

Based on these energy band diagrams the CuBi_2_O_4_/TiO_2_/RuO_*x*_ photoelectrode is also expected to contain a relatively small band offset of approximately 0.3 eV between CuBi_2_O_4_ and TiO_2_. Therefore, assuming negligible formation of interface dipoles, the performance of the CuBi_2_O_4_/TiO_2_/RuO_*x*_ photoelectrode is expected to be comparable to CuBi_2_O_4_/CdS/TiO_2_/RuO_*x*_ and higher than the CuBi_2_O_4_/BiVO_4_/TiO_2_/RuO_*x*_ heterojunction system. As shown in Fig. 2a, this is clearly not the case, suggesting that additional factors aside the band offsets probably play an important role in the heterojunction systems. One of these factors, common in the case of heterojunctions, is the formation of intermixed interface layers, which may serve as hole or electron traps and hinder charge transfer.

### Surface photovoltage analysis

To understand the relation between the charge transfer kinetics in the various heterojunctions and their PEC performance, modulated surface photovoltage (SPV) spectroscopy in the fixed capacitor arrangement was utilized.^26^ Aside from its very high sensitivity, modulated SPV spectroscopy can provide information about fast and slow (or retarded) processes in relation to the modulation period. Modulated SPV spectra are measured with double-phase lock-in amplifiers. The in-phase (*x*) and phase-shifted by 90° (*y*) signals are related to the fast and slow responses, respectively. In general, the response times of the SPV are much shorter (or longer) than the modulation period after switching on and off illumination if *y* (or *x*) are equal to 0. Furthermore, positive (or negative) *x*-signals are related to preferential separation of photogenerated electrons towards the bulk (or surface) of the absorber. The sign of the *y*-signal in relation to the sign of the *x*-signal gives information about the preferential direction of trapped charge carriers. If the *x*- and *y*-signals are of opposite sign, the direction of separated trapped charge carriers is similar to that of fast charge separation and relaxation. In contrast, if the *x*- and *y*-signals have the same sign, the directions of separated charge carriers are opposite for the dominating fast and slow processes (see Fig. S11† for a schematic illustration, and for more detailed explanations also paragraph 2.3.4. in ref. 26 or the ESI in ref. 42).

**Fig. 4:**
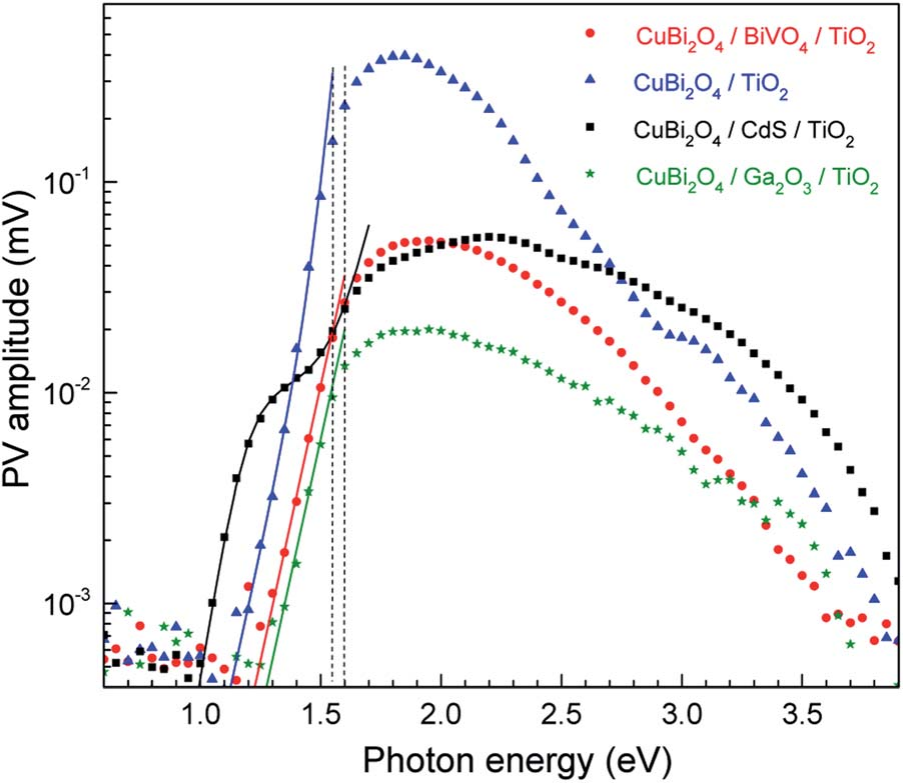
Amplitude spectra of CuBi_2_O_4_/CdS/TiO_2_, CuBi_2_O_4_/BiVO_4_/TiO_2_, CuBi_2_O_4_/TiO_2_ and CuBi_2_O_4_/Ga_2_O_3_/TiO_2_ (black squares, red circles, blue triangles and green stars, respectively). The solid green and red, blue, and black lines are fits below the band gap with one exponential term, the sum of two exponential terms, and the sum of a logarithmic growth and an exponential function for CuBi_2_O_4_/Ga_2_O_3_/TiO_2_ and CuBi_2_O_4_/BiVO_4_/TiO_2_, CuBi_2_O_4_/TiO_2_, and CuBi_2_O_4_/CdS/TiO_2_, respectively. The two vertical dashed lines mark the approximate band gap range of CuBi_2_O_4_.

To start, in order to compare between the different samples in terms of their general SPV response (not separated to slow and fast components), the *x*- and *y*-signals can be converted into amplitudes. The amplitude is defined as the square root of the sum of the squared *x*- and *y*-signals. The amplitude spectra of the CuBi_2_O_4_/CdS/TiO_2_, CuBi_2_O_4_/BiVO_4_/TiO_2_, CuBi_2_O_4_/TiO_2_ and CuBi_2_O_4_/Ga_2_O_3_/TiO_2_ systems are presented on a semi-log scale in Fig. 4. The extracted band gap of CuBi_2_O_4_, which is determined close to where the SPV deviates from exponential dependance, amounts to 1.55–1.6 eV, in agreement with our previous report.^5^ When comparing the maximum measured SPV amplitudes, we find that there is no direct correlation between the maximum SPV signal and the PEC performance, *i.e.* for the CuBi_2_O_4_/TiO_2_ sample, the maximum SPV amplitude is the highest, however for this sample very low photocurrent values were obtained compared to the samples containing CdS or BiVO_4_ buffer layers (see Fig. 2a). Analysis of the amplitude signals below the bandgap can yield valuable information regarding tail states and other defect states in the bulk of the absorber as well as in the interfaces with the different protection layers. If assuming that charge separation is caused only by directed transport of mobile charge carriers in delocalized states, and the SPV measurements are performed in the low-signal case and under homogeneous absorption within the charge separation and/or diffusion lengths, and the *x*- and *y*-signals do not change their signs within the corresponding spectral range, an exponential increase of the amplitude near the band gap or the first derivative of a logistic growth function are directly related to the exponential distribution of states near the valence (or conduction) band edge or to the distribution of deep defect states, respectively, from which photogeneration into delocalized states takes place.

Thus, under the above assumptions, we find the following fitting functions were needed to obtain a good fit, depending on the sample:1
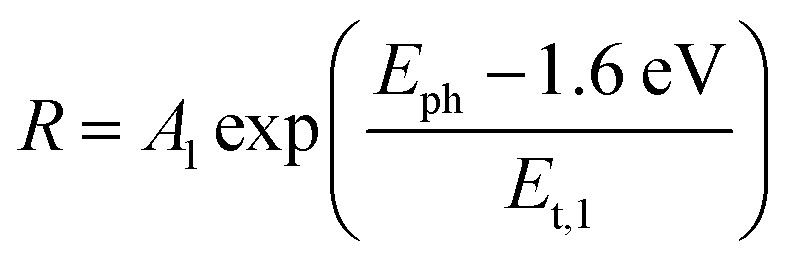
2
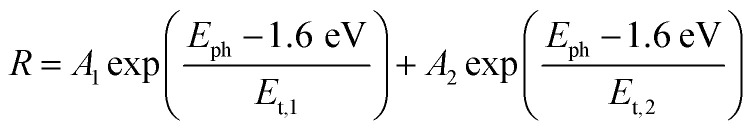
3
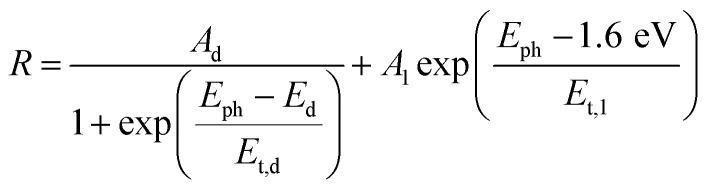


For the CuBi_2_O_4_/BiVO_4_/TiO_2_ and CuBi_2_O_4_/Ga_2_O_3_/TiO_2_ samples, a single exponential term (eqn (1)) was sufficient. For the CuBi_2_O_4_/TiO_2_, a sum of two exponential terms was required (eqn (2)), and for the CuBi_2_O_4_/CdS/TiO_2_ a sum of a logarithmic growth and an exponential function was required (eqn (3)). An identical band gap of 1.6 eV was used for all fits of the increasing part of the amplitude spectra. The main, common exponentials (eqn (1)) were characterized by the same tail energy (*E*_t,1_) equal to 83 meV in all equations (we note that *E*_t_ is related, yet not equal to the so-called Urbach tail which is experimentally obtained from optical absorption). This gives evidence that *E*_t,1_ is related to disorder-induced defect states near the band gap of the bulk of CuBi_2_O_4_ and that this disorder was not affected by the deposition of the different protection layers on top. Furthermore, the values of *A*_1_ amounted to 20, 36, 130 and 15.5 μV for CuBi_2_O_4_/Ga_2_O_3_/TiO_2_, CuBi_2_O_4_/BiVO_4_/TiO_2_, CuBi_2_O_4_/TiO_2_ and CuBi_2_O_4_/CdS/TiO_2_, respectively. Therefore, the strongest charge separation from defect states near the band gap of CuBi_2_O_4_ appeared in CuBi_2_O_4_/TiO_2_.

The values of *E*_t,2_ and *A*_2_ were 35 meV and 600 μV, respectively. The fact that a second exponential term was required for fitting the increase of the SPV amplitude below the band gap of CuBi_2_O_4_ in CuBi_2_O_4_/TiO_2_ gives evidence for the formation of an interface region with efficient absorption and modulated charge separation at reduced disorder near the band gap of CuBi_2_O_4_.

The logarithmic growth function in eqn (3) is related to the excitation and separation of mobile charge carriers due to absorption in relatively deep defect states (where *E*_d_ is denotes the maximum DOS of the defect distribution within the bandgap). The obtained *E*_d_ value amounts to 1.19 eV, about 400 meV within the bandgap of the CuBi_2_O_4_, in agreement with the deep defects observed in our previous work for this type of junction.^5^ These deep defect states were specific for the CuBi_2_O_4_/CdS interface since the defect related feature appeared only in the spectrum of CuBi_2_O_4_/CdS/TiO_2_. It can therefore be concluded that partial inter-diffusion took place at the CuBi_2_O_4_/CdS interface leading to the formation of deep interfacial states. In spite of these additional deep defect states that were found only in the CuBi_2_O_4_/CdS/TiO_2_ sample, this sample had the highest PEC performance. Thus, in order to a gain deeper understanding of how the modulated charge separation and recombination processes affect and correlate to the actual PEC performance, it is essential to understand exactly which type of free and/or trapped charge carriers are accumulated at the different interfaces, by performing a detailed analysis of the individual *x* and *y* components of the SPV signals, as shown next.

**Fig. 5:**
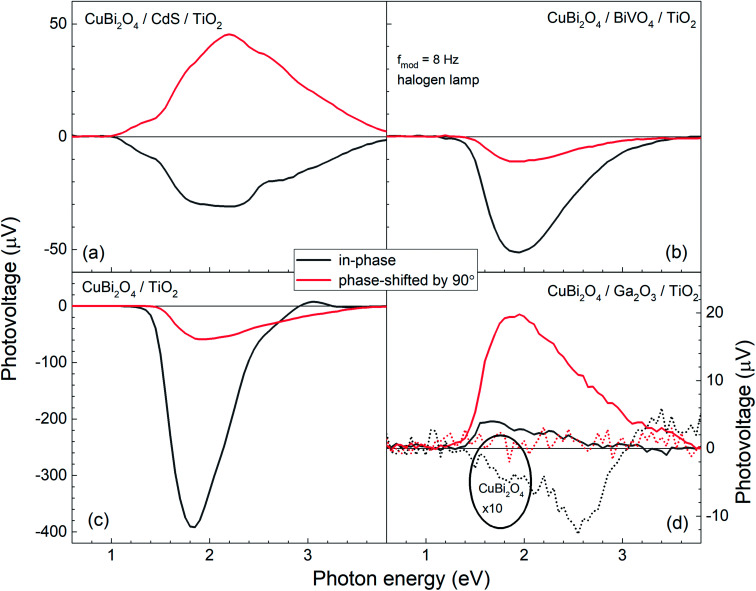
Modulated SPV spectra of the in-phase and phase-shifted by 90° signals (black and red solid lines, respectively) for the CuBi_2_O_4_/CdS/TiO_2_, CuBi_2_O_4_/BiVO_4_/TiO_2_, CuBi_2_O_4_/TiO_2_ and CuBi_2_O_4_/Ga_2_O_3_/TiO_2_, samples ((a)–(d), respectively). For comparison, the thin dashed black and red lines in (d) represent the measured in-phase and phase-shifted by 90° signals for a bare CuBi_2_O_4_ layer with uncontrolled surface.

Fig. 5 shows the modulated SPV spectra of the in-phase and phase-shifted by 90° signals for the CuBi_2_O_4_/CdS/TiO_2_, CuBi_2_O_4_/BiVO_4_/TiO_2_, CuBi_2_O_4_/TiO_2_ and CuBi_2_O_4_/Ga_2_O_3_/TiO_2_, samples. Starting with the in-phase signals, that give indication into which type of free charge carriers accumulate closer to the sample's surface, it is found that in the absorption range of CuBi_2_O_4_, the signs of the in-phase signals where negative for CuBi_2_O_4_/CdS/TiO_2_, CuBi_2_O_4_/BiVO_4_/TiO_2_, CuBi_2_O_4_/TiO_2_ and bare CuBi_2_O_4_ with an uncontrolled surface. This finding gives evidence to separation of electrons towards the surface (as expected for a p-type absorber with an electron-selective contact or a p-type semiconductor with a depletion region near the surface), which is favorable for a photoreduction reaction such as proton reduction to take place at the surface. Incidentally, the signals of the bare CuBi_2_O_4_ layer with an uncontrolled surface were extremely low (only on the order of a μV) in comparison to the CuBi_2_O_4_/CdS/TiO_2_, CuBi_2_O_4_/BiVO_4_/TiO_2_, and CuBi_2_O_4_/TiO_2_ samples. This suggests that without an appropriate charge extraction layer, the surface of the CuBi_2_O_4_ is not passivated, and/or the charge separation efficiency within the CuBi_2_O_4_ depletion region is rather low. In contrast, for the CuBi_2_O_4_/Ga_2_O_3_/TiO_2_ sample, the sign of the in-phase signals was positive and only on the order of several μV. The positive sign of the in-phase signals indicates separation of holes towards the surface, however due to the very low signal, the charge separation efficiency in this case is quite low (as shown schematically in Fig. 7d), explaining the poor PEC performance of the CuBi_2_O_4_/Ga_2_O_3_/TiO_2_/RuO_*x*_ heterojunction as a photocathode (Fig. 2a). This observed behavior is probably due to the unfavorable band alignment already discussed and shown in Fig. 3c.

With regards to the phase-shifted by 90° signals, positive phase-shifted by 90° signals were found for CuBi_2_O_4_/CdS/TiO_2_ and CuBi_2_O_4_/Ga_2_O_3_/TiO_2_, suggesting that for these two samples, electrons that were separated towards the external surface were trapped in the range of the CuBi_2_O_4_/CdS or the CuBi_2_O_4_/Ga_2_O_3_ interfaces. The electron traps in the CuBi_2_O_4_/CdS interface can probably be attributed to the deep defect states seen in the amplitude spectrum of the CuBi_2_O_4_/CdS/TiO_2_ sample in the energy region of 1–1.5 eV. In contrast, the signs of the phase-shifted by 90° signals were negative for CuBi_2_O_4_/BiVO_4_/TiO_2_ and CuBi_2_O_4_/TiO_2_, *i.e.* holes were separated towards the external surface and trapped. This finding suggests that for these two heterojunction types, predominant trap states for holes were formed at CuBi_2_O_4_/BiVO_4_ and CuBi_2_O_4_/TiO_2_ interfaces, as shown schematically in Fig. 5b and c. Such an accumulation of trapped holes at the interface with CuBi_2_O_4_, compared to trapped electrons in the case of CuBi_2_O_4_/CdS, is much more detrimental for the PEC performance since it will lead to an increase in the total number of photogenerated electrons that will (non-radiatively) recombine. This explains the lower observed photocurrents for CuBi_2_O_4_/TiO_2_ heterojunction in Fig. 2a compared to CuBi_2_O_4_/CdS/TiO_2_.

**Fig. 6:**
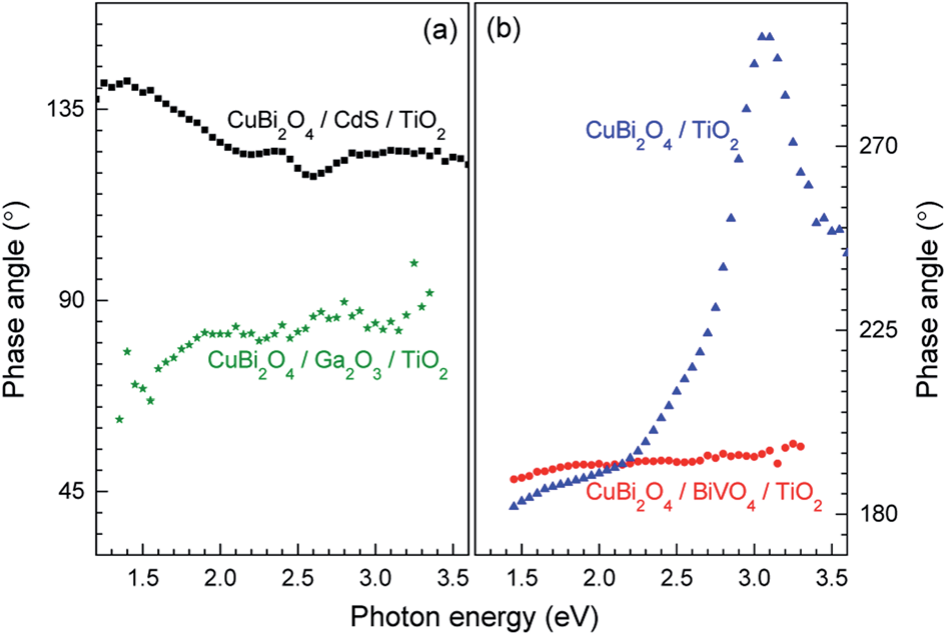
Spectra of the phase angles of CuBi_2_O_4_/CdS/TiO_2_ and CuBi_2_O_4_/Ga_2_O_3_/TiO_2_ ((a), black squares and green stars, respectively) and of CuBi_2_O_4_/BiVO_4_/TiO_2_ and CuBi_2_O_4_/TiO_2_ ((b), red circles and blue triangles, respectively).

Although in both CuBi_2_O_4_/BiVO_4_/TiO_2_ and CuBi_2_O_4_/TiO_2_ a combination of negative in-phase and phase-shifted by 90° signals was observed, the PEC performance of the CuBi_2_O_4_/TiO_2_ sample was extremely poor compared to the sample with the BiVO_4_ buffer layer, which requires explanation. This behavior can be attributed to an additional, pronounced difference that is observed with and without the BiVO_4_ buffer layer: for the CuBi_2_O_4_/TiO_2_ sample, in the region between 2.5–3.5 eV, the phase-shifted by 90° signal becomes larger than the in-phase signal, and the in-phase signal changes sign. Such pronounced qualitative differences can be analyzed quantitively by considering the so-called phase angle of the signal. The in-phase (*x*) and quadrature (*y*) components of the signal can be converted into the phase angle, which is defined as the arctan of the ratio between the *y*- and *x*-signals. Since the behavior of the *x*- and *y*-signals depends sensitively on transport and charge transfer processes, changes in the phase angle can provide information about changes in dominating processes in modulated charge separation. Spectra of the phase angles of CuBi_2_O_4_/CdS/TiO_2_, CuBi_2_O_4_/Ga_2_O_3_/TiO_2_, CuBi_2_O_4_/BiVO_4_/TiO_2_ and CuBi_2_O_4_/TiO_2_ are given in Fig. 6.

For CuBi_2_O_4_/CdS/TiO_2_, the phase angles changed from about 140° at 1.4 eV to 119° at 2.6 eV and to 124° at photon energies above 2.85 eV. Between 2.2 and 2.6 eV, the phase angle peaked slightly from 124° to 125° at 2.4 eV (band gap of CdS), decreased to the minimum of 119° at 2.6 eV, and then increased to its saturation value after that. Therefore, since those changes were small, absorption of light by defect states near the band gap of CdS and fundamental absorption in CdS led only to little modification in trapping.

For CuBi_2_O_4_/Ga_2_O_3_/TiO_2_ and CuBi_2_O_4_/BiVO_4_/TiO_2_, the phase angles changed from about 75° to 86° and from about 189° to 196°, respectively. No well-defined signatures could be found within those weak changes. Therefore, deposition of Ga_2_O_3_ or BiVO_4_ onto CuBi_2_O_4_ does not result in the formation of additional transitions that influence the trapping dynamics.

**Fig. 7:**
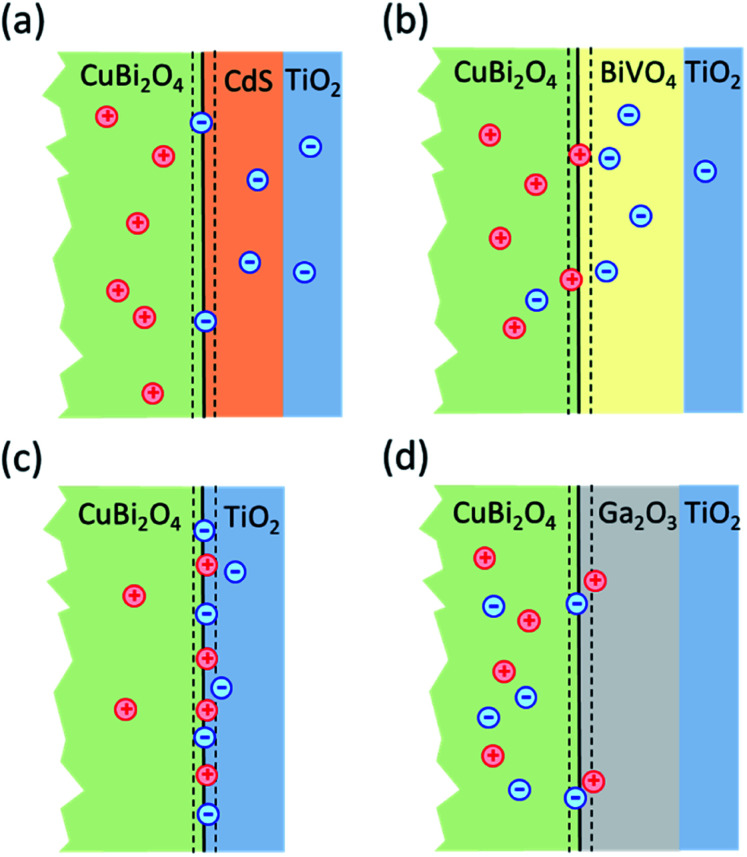
Schematic model illustrating the distribution of charge carriers in the different heterojunctions upon illumination with white light: (a) CuBi_2_O_4_/CdS/TiO_2_; (b) CuBi_2_O_4_/BiVO_4_/TiO_2_; (c) CuBi_2_O_4_/TiO_2_ and (d) CuBi_2_O_4_/Ga_2_O_3_/TiO_2_.

In contrast, drastic changes were observed in the spectrum of the phase angles for CuBi_2_O_4_/TiO_2_. At photon energies between 1.45 and 2.15 eV, the phase angle increased from about 182° to 192°. The fact that the phase angles were so close to 180° means that the forward and backward electron transfer was the fastest at the CuBi_2_O_4_/TiO_2_ interface. Between 2.15 and 2.7 eV, the phase angle increased strongly to 224° which means that the electron transfer became very slow in relation to the modulation period, *i.e.* strong electron trapping set in. The very strong change of the phase angles up to values exceeding 270° (296° at 3.05–3.10 eV) shows that a large change in the modulated charge separation occurs at these photon energies. Specifically, a phase angle around 270° indicates that the photo-generated holes move towards the external surface and/or that the photo-generated electrons move towards the CuBi_2_O_4_/TiO_2_ interface. This explains the very poor performance of the CuBi_2_O_4_/TiO_2_ photocathode sample without a buffer layer. At photon energies 3.1 eV, the phase angle changed towards lower values (244° at 3.6 eV) giving evidence for an increased influence of the separation of electrons photo-generated in TiO_2_ towards the external surface. The very strong change of the phase angles for CuBi_2_O_4_/TiO_2_ can only be interpreted by the formation of defect states within the band gap of TiO_2_ near the CuBi_2_O_4_/TiO_2_ interface, probably caused by inter-diffusion and/or partial reduction of TiO_2_ by transferred and trapped holes. This notable difference in the dynamics and distribution of the charge carriers with and without the BiVO_4_ buffer layer, illustrated in Fig. 7b and c, demonstrates the need to prevent direct contact between the CuBi_2_O_4_ and the TiO_2_.

Based on the analysis of the SPV results and the correlation found with the PEC performance of the different heterojunctions, a schematic model illustrating the distribution of charge carriers in the different heterojunctions upon illumination with white light is shown in Fig. 7. The CuBi_2_O_4_/CdS/TiO_2_ sample (Fig. 7a) shows the highest PEC performance, which we attribute to the uniform separation of free and trapped electrons toward the TiO_2_ surface. When the CdS layer is replaced with BiVO_4_ (Fig. 7b), interfacial hole traps are formed at the CuBi_2_O_4_/BiVO_4_ interface, enhancing non-radiative recombination of electron–hole pairs and resulting in lower photocurrents. In the case of direct contact between the CuBi_2_O_4_ and TiO_2_ (Fig. 7c), large defect-related SPV signals are observed, and a large majority of the photogenerated electrons and holes are trapped at the CuBi_2_O_4_/TiO_2_ interface, resulting in poor PEC performance. For the case of CuBi_2_O_4_/Ga_2_O_3_/TiO_2_ (Fig. 7d), the charge separation efficiency is quite low compared to the other heterojunctions, probably due to the large energetic barriers due to the higher conduction band of Ga_2_O_3_ shown in Fig. 3. The charge separation direction is not favorable for photocathodic reactions, explaining the very poor PEC performance observed. Overall, based on the modulated SPV results, it can be concluded that one of the most notable differences between the different heterojunctions is the lack of trapped holes in the heterojunction containing the CdS buffer layer. Hence, we conclude that trapped holes at the interface with the CuBi_2_O_4_ are the main cause for interfacial recombination and poor photocurrents.

## Conclusions

In this work, we showed that photocurrents generated from bare, unprotected CuBi_2_O_4_ photocathodes were mainly due to photo-corrosion of CuBi_2_O_4_ and not from H_2_ production, as claimed by many reports on Cu-based photocathodes. We investigated the influence of different buffer layers between the CuBi_2_O_4_ absorber and an n-type TiO_2_ film that serves as a protection layer. We found that a CdS buffer layer in combination with the ALD-deposited TiO_2_ protection layer and RuO_*x*_ co-catalyst layer yielded a stable photoelectrode with the highest photocurrent density and faradaic efficiency for the hydrogen evolution reaction. In contrast, neither high photocurrent nor efficient hydrogen evolution was obtained for CuBi_2_O_4_/Ga_2_O_3_/TiO_2_, CuBi_2_O_4_/BiVO_4_/TiO_2_ and CuBi_2_O_4_/TiO_2_ heterojunctions. However, band alignment considerations alone cannot explain the observed trends in photoelectrochemical performance. Therefore, modulated surface photovoltage measurements were used to investigate the mechanism governing the charge transport in these heterojunctions. The modulated SPV results strongly correlated with the observed trend of the photoelectrochemical performance and revealed the formation of different interfacial states, depending on the CuBi_2_O_4_/buffer layer junction. Most importantly, it revealed which type of charge carrier was trapped at the interface with the CuBi_2_O_4_ suggesting that trapping of holes near the interface strongly limits the photo-electrochemical performance of the heterojunctions.

## Author contributions

The manuscript was written through contributions of all authors. All authors have given approval to the final version of the manuscript.

## Conflicts of interest

There are no conflicts to declare.

## Supplementary Material

SC-011-D0SC03030A-s001
