# Supplementary material for: Revealing the relationship between photoelectrochemical performance and interface hole trapping in CuBi2O4 heterojunction photoelectrodes
Source: Chem Sci. 2020 Sep 14;11(41):11195–204. doi: 10.1039/d0sc03030a (PMC8162275; doi:10.1039/d0sc03030a)
Supplement: SC-011-D0SC03030A-s001 [file SC-011-D0SC03030A-s001.pdf]

Electronic Supplementary Material (ESI) for Chemical Science.
This journal is © The Royal Society of Chemistry 2020

Supporting Information

Revealing the Relationship between Photoelectrochemical Performance and Interface Hole Trapping in $CuBi_2O_4$ Heterojunction Photoelectrodes

Angang Song,[ab] Igal Levine,[c] Roel van de Krol,[ab] Thomas Dittrich[c*] and Sean P. Berglund[a*]

a. Institute for Solar Fuels, Helmholtz-Zentrum Berlin für Materialien und Energie GmbH, Hahn-Meitner-Platz 1, 14109 Berlin, Germany.

b. Institut für Chemie, Technische Universität Berlin, Straße des 17. Juni 124, 10623 Berlin, Germany.

c. Institute for Silicon Photovoltaics, Helmholtz-Zentrum Berlin für Materialien und Energie GmbH, Kekuléstr. 5, 12489 Berlin, Germany.

Corresponding Authors:

*T.D. Email: dittrich@helmholtz-berlin.de

*S.P.B. Email: sean.berglund@gmail.com

Experimental methods

Fabrication of $CuBi_2O_4$ photocathodes

The typical synthesis procedure for a $CuBi_2O_4$ photocathode is based on our previous work. [1, 2] Briefly, the $Bi(NO_3)_3$ precursor was first sprayed onto the FTO substrate at a deposition temperature of 450 °C to form a bismuth oxide layer. Then the $Cu(NO_3)_2$ precursor was sprayed successively on top of the bismuth oxide layer at 450 °C and a gradient self-doped $CuBi_2O_4$ thin film was formed by diffusion of copper into the film. The thickness of the $CuBi_2O_4$ thin film was approximately 280 nm.

Deposition of CdS buffer layer

A CdS buffer layer was deposited on top of the $CuBi_2O_4$ film using chemical bath deposition (CBD). In a typical synthesis procedure, a beaker containing 150 mL of stirred ultrapure water was heated inside a water bath. When the temperature of the solution reached 65 °C, 22 mL of 15 mM $CdSO_4$ solution was added to the bath. Then 22 mL of NH_4OH solution was added to the chemical bath followed by immersion of the $CuBi_2O_4$ films into the solution for 15 min. The CdS-coated $CuBi_2O_4$ films were then rinsed thoroughly with water and dried in an oven at 120 °C. The thickness of the CdS film was approximately 100 nm.

Deposition of $BiVO_4$ buffer layer

$BiVO_4$ buffer layers were prepared using spray pyrolysis. The precursor solution was made by dissolving 4 mM $Bi(NO_3)_3{\cdot}5H_2O$ (98%, Alfa Aesar) in acetic acid (98%, Sigma-Aldrich) and adding an equimolar amount of vanadium in the form of $VO(AcAc)_2$ (99%, Alfa Aesar) dissolved in absolute ethanol (Sigma-Aldrich). Each spray cycle consisted of 5 s of spray time and 55 s of delay time to allow for solvent evaporation, and a total of 100 cycles were used to deposit the $BiVO_4$ films. More details can be found in previous reports. [3, 4] The thickness of the resulting $BiVO_4$ film was approximately 100 nm.

Deposition of Ga_2O_3 buffer layer

Ga_2O_3 was deposited by atomic layer deposition (ALD). Before being placed inside the ALD reaction chamber, the $CuBi_2O_4$ samples were rinsed thoroughly with deionized water and dried under a stream of N_2. The deposition was carried out at 170 °C using sequential pulses of Tris (dimethylamido) gallium (III) 98% (precursor temperature: 130 °C), followed by a purge, O_2-plasma treatment, and another purge. O_2-plasma treatments were done using an RF power of 2800 W with 40 sccm Ar and 100 sccm O_2. The thickness of the $CuBi_2O_4$ thin film was approximately 280 nm. The thickness of amorphous Ga_2O_3 thin film was about 25 nm, which was determined from ellipsometric measurements on a piece of silicon witness wafer.

Deposition of TiO_2 protection layer

TiO_2 was deposited by ALD. Before deposition, the $CuBi_2O_4$ sample was rinsed thoroughly with deionized water and dried under a stream of N_2 before placing it in the ALD reaction chamber. The deposition was carried out at 120 °C using sequential pulses of tetrakis (dimethylamino) titanium (precursor temperature: 85 °C) and H_2O (precursor temperature: 25 °C). The thickness of amorphous TiO_2 thin film was about 20 nm, which was determined from ellipsometric measurements on a piece of silicon witness wafer.

Deposition of Co-catalysts

The ruthenium oxide (RuO_x) co-catalyst was photo-electrodeposited onto the $CuBi_2O_4/Ga_2O_3/TiO_2$, $CuBi_2O_4/BiVO_4/TiO_2$, $CuBi_2O_4/CdS/TiO_2$ and $CuBi_2O_4/TiO_2$ samples from an aqueous solution of 1.17 mM $KRuO_4$ in 25 mL deionized water, using a constant current of −0.03 mA/cm^2 for 10 min with constant illumination from the solar simulator (100 mW/cm^2). The photo-electrodeposition was carried out in three-electrode configuration with a platinum counter electrode and an Ag/AgCl electrode (saturated KCl) as the reference electrode.

Material Characterization

The morphology of the films was analyzed using a LEO GEMINI 1530 field emission scanning electron microscope (FESEM) operated at an acceleration voltage of 7 kV. Elemental analysis using X-ray photoelectron spectroscopy (XPS) was carried out with a monochromatic Al K_α X-ray source (1486.74 eV, Specs Focus 500 monochromator) and a hemispherical analyzer (Specs Phoibos 100) in an ultrahigh vacuum system (base pressure ~10^{-8} mbar). Ultraviolet photoelectron spectroscopy (UPS) was conducted using a He I source (E = 21.21 eV) with the same hemispherical analyzer as in the XPS measurement. In order to remove possible surface contamination all films were cleaned using an oxygen plasma for 5 min prior to the measurement. The plasma was deployed using a radio frequency plasma generator (MANTIS(R)) with an oxygen partial pressure of 4 x 10^{-5} mbar (gas purity 99.999 %) and a workload of 200 W.

Characterization by modulated surface photovoltage spectroscopy

Modulated surface photovoltage measurements were performed in the fixed capacitor arrangement in air. A halogen lamp with a quartz prism monochromator (SPM2) was used for excitation. The modulation frequency was 8 Hz. The in-phase (X) and phase-shifted by 90° (Y) signals were measured with a double-phase lock-in amplifier (EG&G5210). More details about the setup, measurement regime, and data analysis are given in a recently published book.[5]

Photoelectrochemical Characterization

Three-electrode photoelectrochemical measurements were performed under the control of a potentiostat (EG&G Princeton Applied Research 273A) with the samples connected as the working electrode, a Pt wire as the counter electrode, and an Ag/AgCl electrode (saturated KCl) as the reference electrode. Measurements were performed in a 0.3 M K_2SO_4 and 0.2 M phosphate buffer (pH 6.8), which was checked by a pH meter (OAKTON). The illumination source was a WACOM super solar simulator (Model WXS-50S-5H, class AAA), which was calibrated to closely resemble the AM1.5 global spectrum at 100 mW/cm^2. All the measured potentials were converted to the reversible hydrogen electrode (RHE) scale using the Nernstian relation:

$$V_{RHE} = V_{Ag/AgCl} + 0.0591 \times pH + 0.197(V)$$

For current vs. voltage and H_2 gas signal vs. voltage measurements, differential electrochemical mass spectrometry (DEMS) measurements was carried out in a thin-electrolyte-layer PEC cell with the photoelectrodes as the working electrode, a Pt wire as the counter electrode, and an Ag/AgCl as the reference electrode. The coupling to the mass spectrometer is achieved via a hydrophobic semipermeable membrane and a differential pumped vacuum system. The illumination source was a 150 W Xe lamp with an AM 1.5G filter. After each mass spectrometer measurement, the electrolyte was exchanged to avoid undesired shifts in the pH.

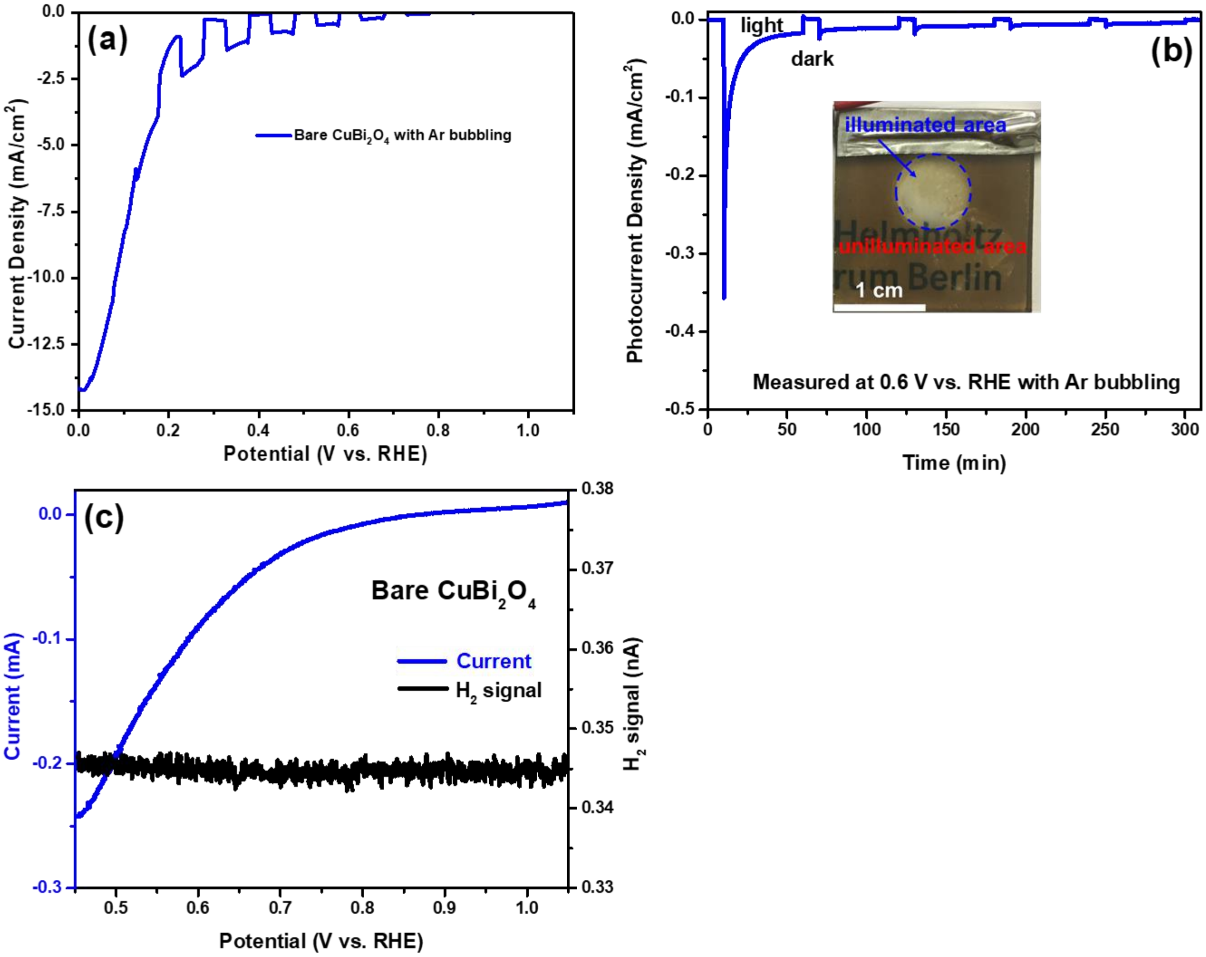

Figure S1. (a) Chopped LSV scans for a 280 nm $CuBi_2O_4$ photocathode. (b) Constant potential measurement at 0.6 V vs RHE for bare $CuBi_2O_4$ photocathode under frontside simulated AM1.5 illumination. (c) DEMS LSV scans for a $CuBi_2O_4$ photocathode with illumination, showing current (blue) and H_2 signal (black). All measurements were performed in three-electrode configuration in 0.3 M K_2SO_4 and 0.2 M phosphate buffer electrolyte (pH 6.8) as the base electrolyte with Ar bubbling.

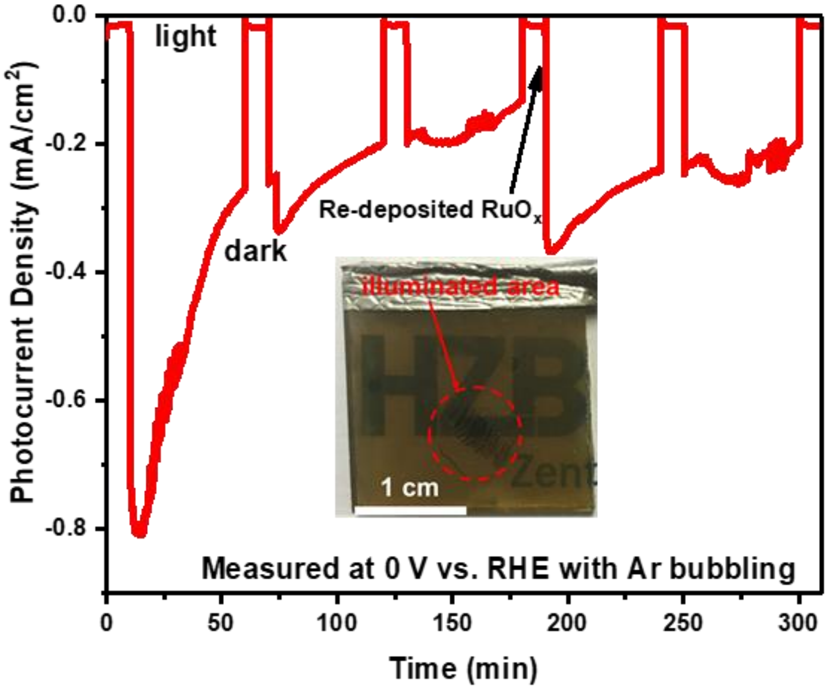

Figure S2. Constant potential measurement at 0 V vs RHE for $CuBi_2O_4/CdS/TiO_2/RuO_x$ photocathode under frontside simulated AM1.5 illumination. The measurement was done in 0.3 M K_2SO_4 and 0.2 M phosphate buffer (pH 6.8) with Ar bubbling.

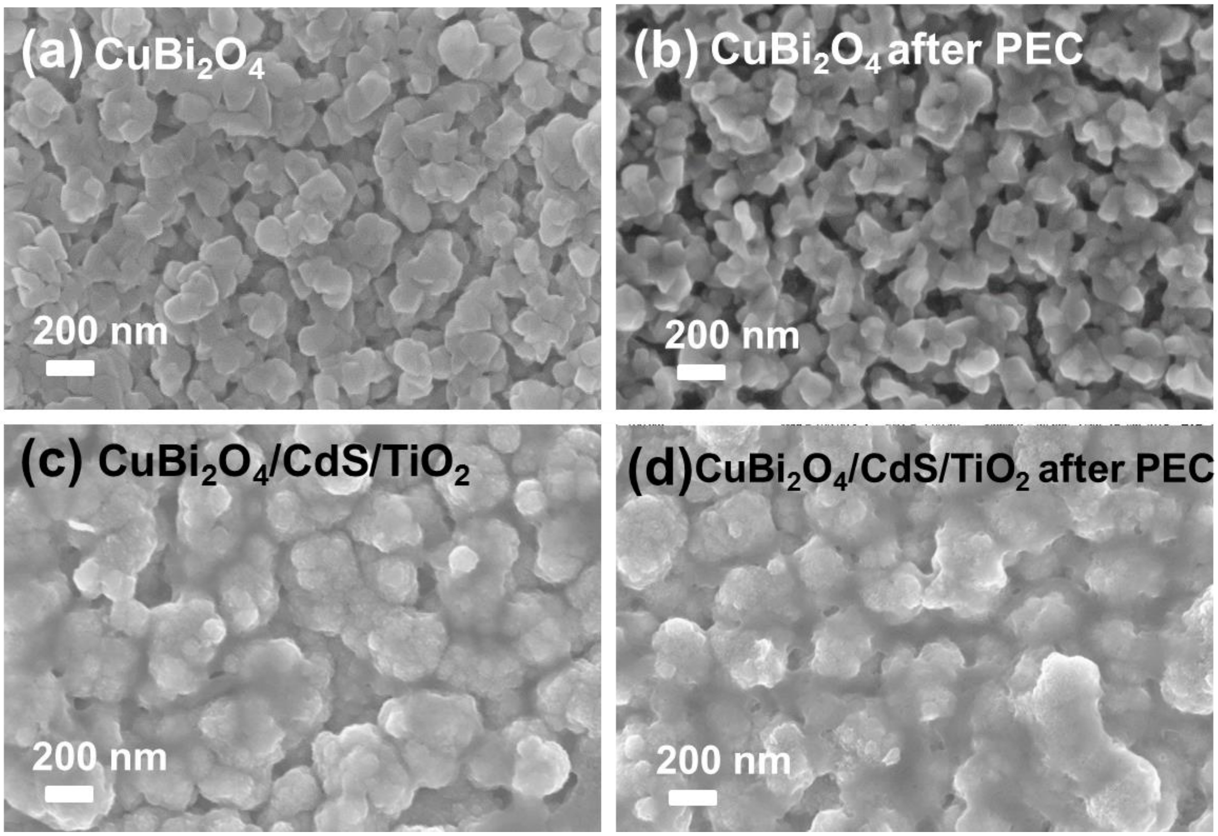

Figure S3. SEM images of a bare $CuBi_2O_4$ photocathode (a) without PEC testing and (b) after PEC testing, (c) a $CuBi_2O_4/CdS/TiO_2$ photoelectrode without PEC testing, and (d) a $CuBi_2O_4/CdS/TiO_2/RuO_x$ photoelectrode after a PEC testing.

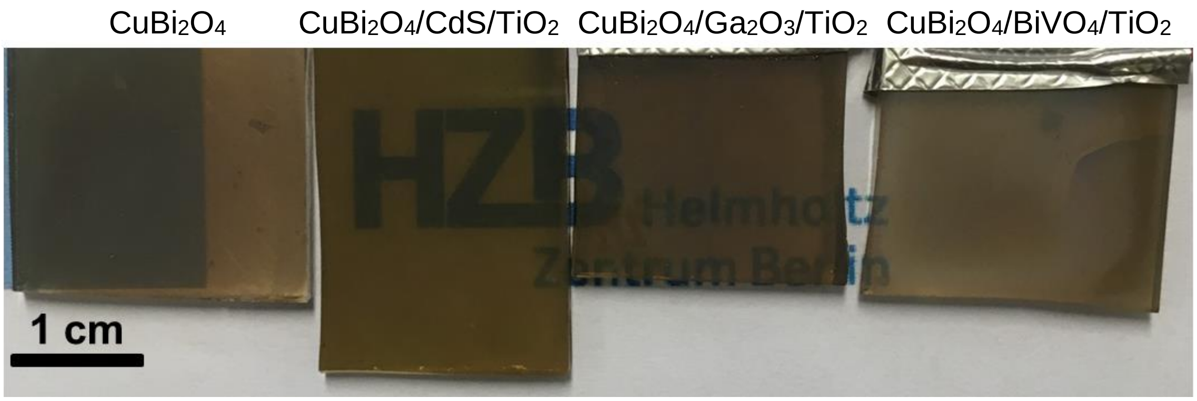

Figure S4. Photographs of bare $CuBi_2O_4$, $CuBi_2O_4/CdS/TiO_2$, $CuBi_2O_4/Ga_2O_3/TiO_2$, and $CuBi_2O_4/BiVO_4/TiO_2$ photoelectrodes on an FTO-coated glass substrate. CBO denotes $CuBi_2O_4$.

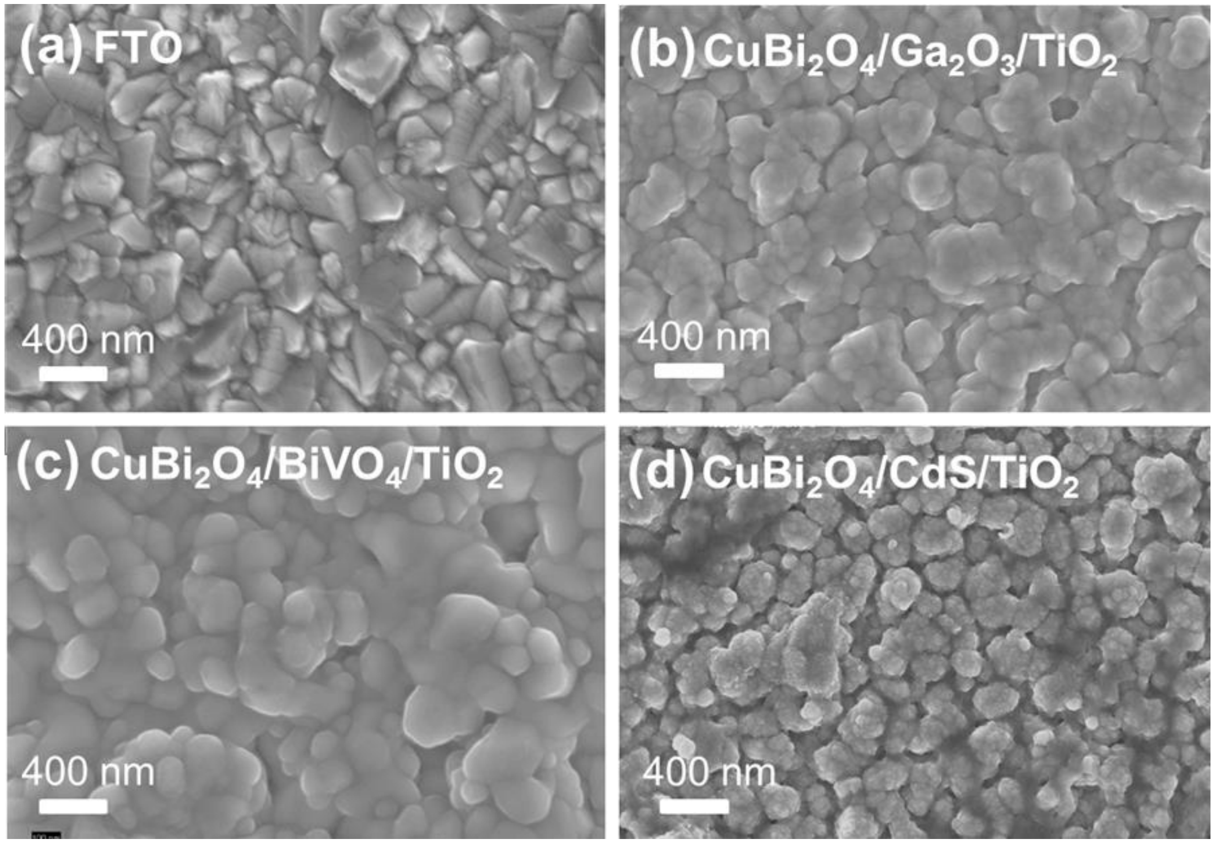

Figure S5. SEM images of a (a) bare FTO substrate, (b) $CuBi_2O_4/Ga_2O_3/TiO_2$, (c) $CuBi_2O_4/BiVO_4/$ TiO_2, (d) $CuBi_2O_4/CdS/TiO_2$ photoelectrodes.

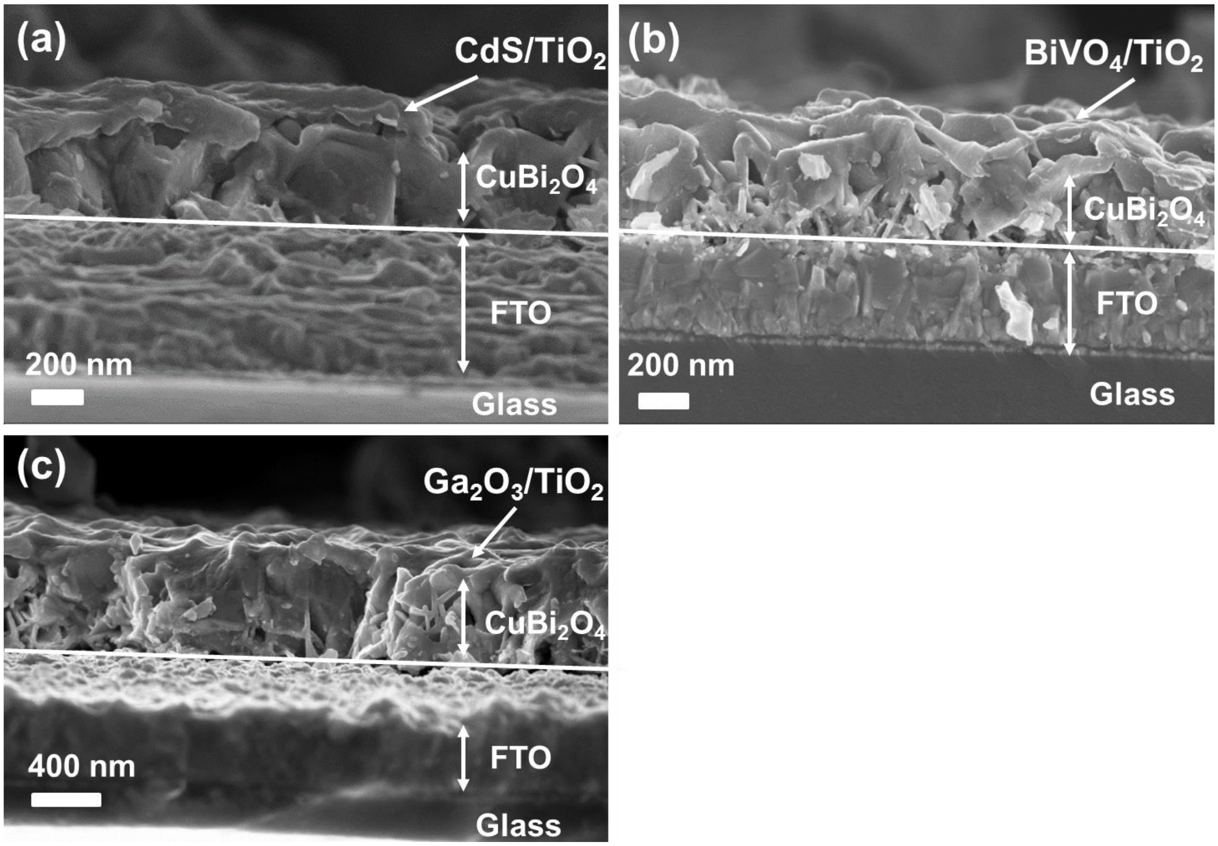

Figure S6. Cross-section SEM images of a (a) $CuBi_2O_4/CdS/TiO_2$, (b) $CuBi_2O_4/BiVO_4/TiO_2$, (c) $CuBi_2O_4/Ga_2O_3/TiO_2$ photoelectrode deposited on an FTO substrate with the different layers indicated.

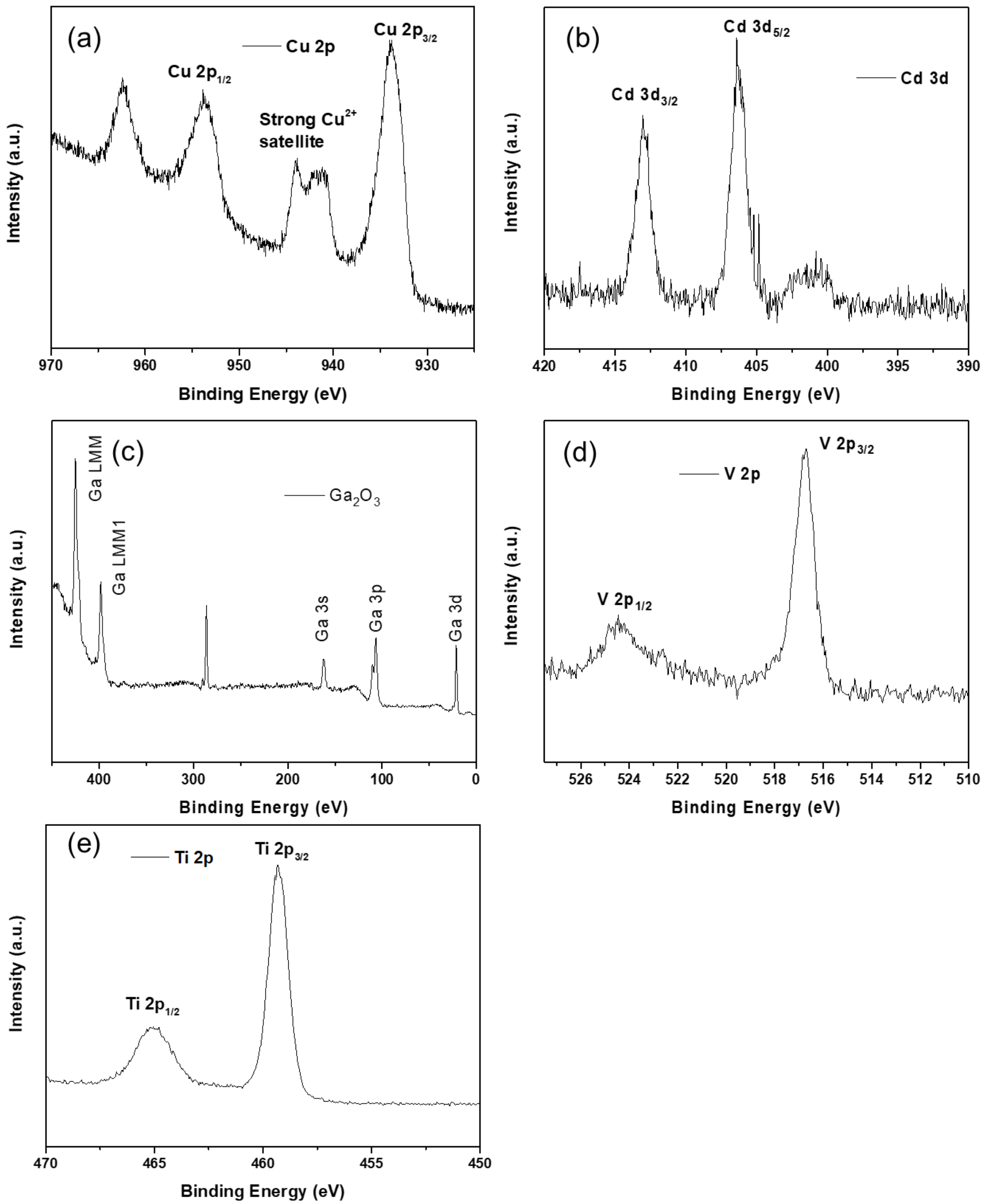

Figure S7. XPS spectra of (a) Cu 2p for bare $CuBi_2O_4$ photocathode, (b) Cd 3d for CdS buffer layer, (c) survey spectra for Ga_2O_3 buffer layer, (d) V 2p for $BiVO_4$ buffer layer and (d) Ti 2p for TiO_2 protective layer.

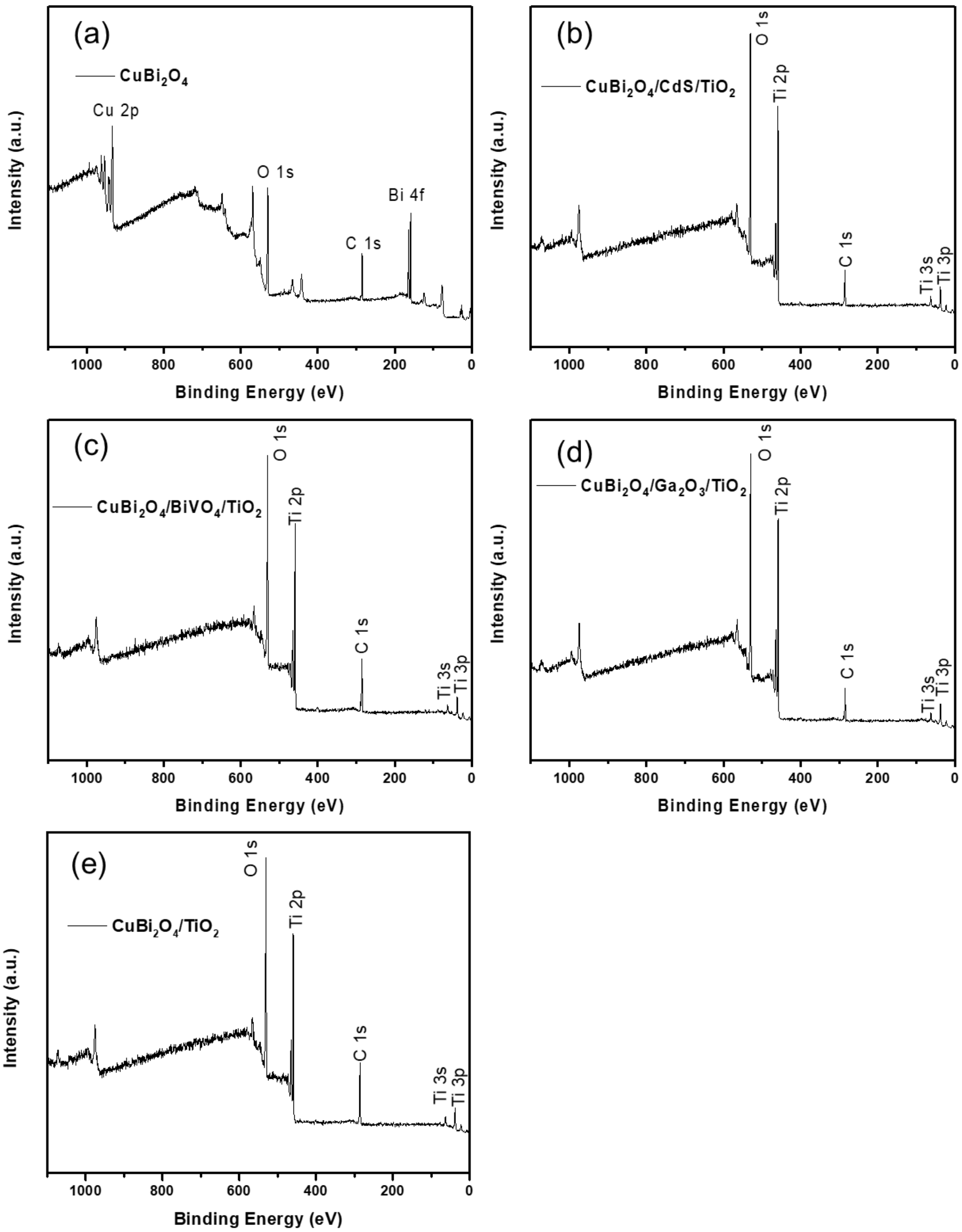

Figure S8. XPS survey spectra of (a) a bare $CuBi_2O_4$ photocathode (b) $CuBi_2O_4$/CdS/TiO_2, (c) $CuBi_2O_4$/$BiVO_4$/TiO_2, (d) $CuBi_2O_4$/Ga_2O_3/TiO_2 photoelectrode, and (e) $CuBi_2O_4$/ TiO_2 photoelectrode.

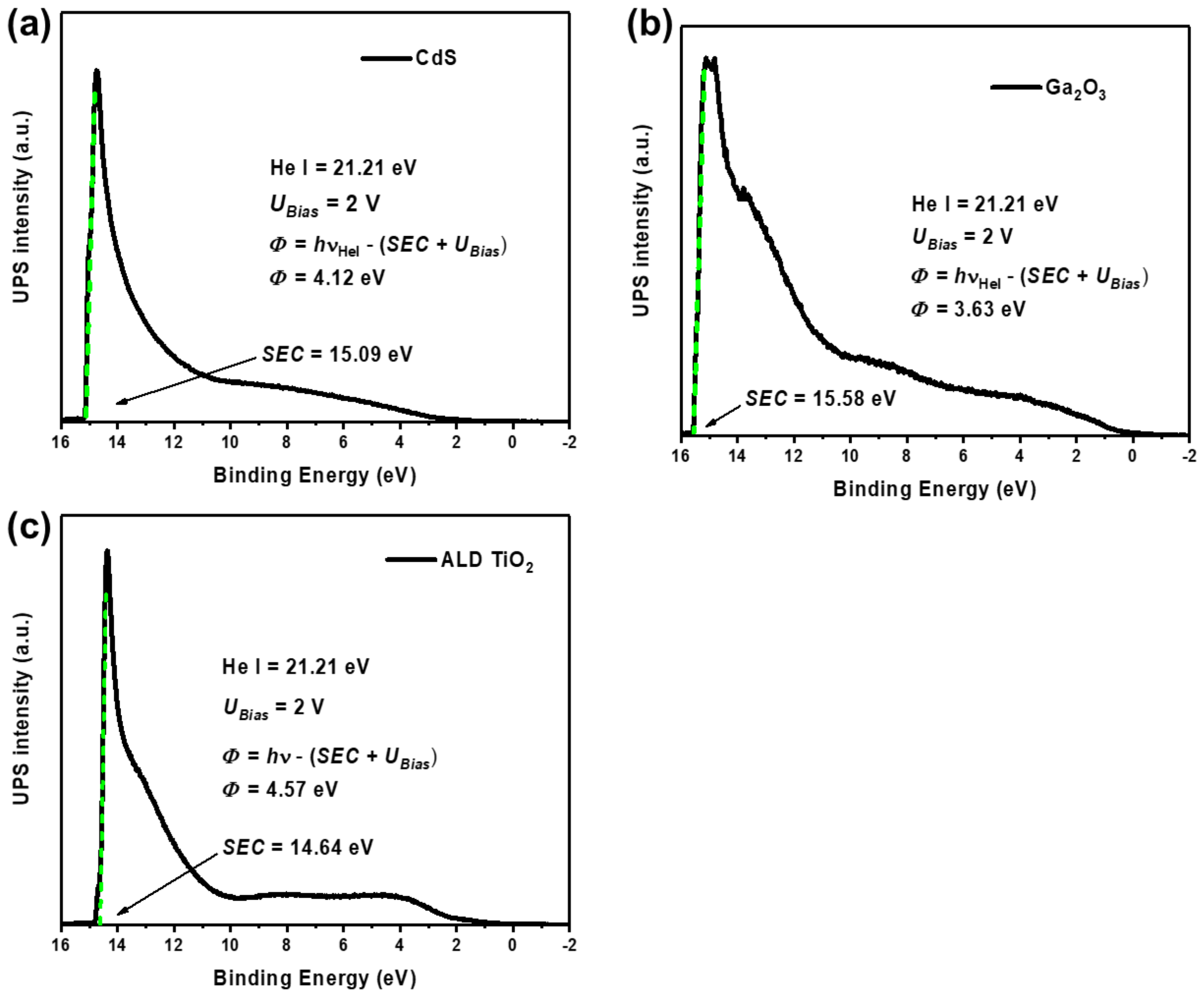

Figure S9. UPS cutoff spectra measured with a 2 V bias for (a) CdS film, (b) Ga_2O_3 film and (c) TiO_2 on FTO substrate.

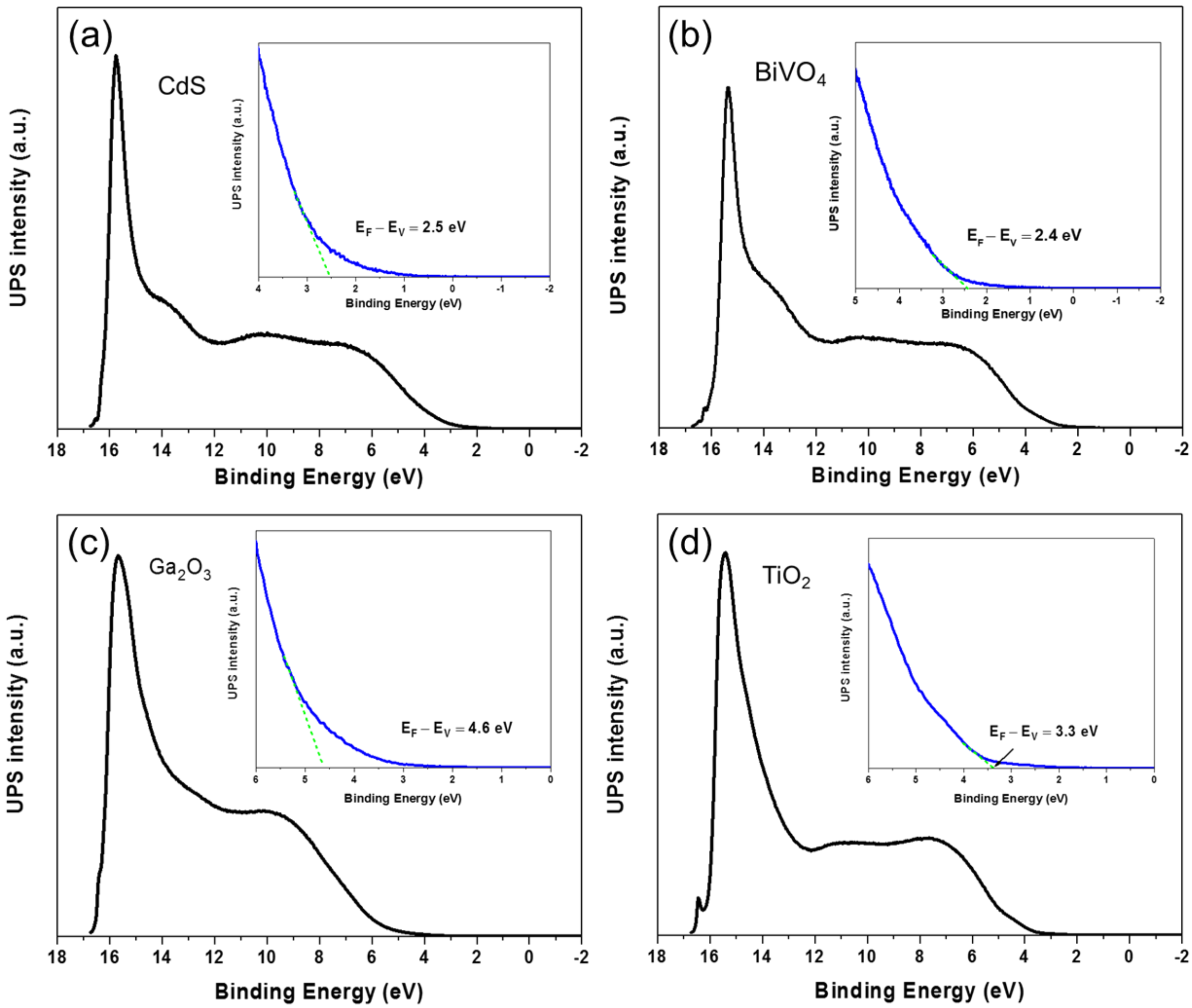

Figure S10. UPS cutoff spectra measured without bias for (a) CdS film, (b) $BiVO_4$ film, (c) Ga_2O_3 film and (d) TiO_2 thin film.

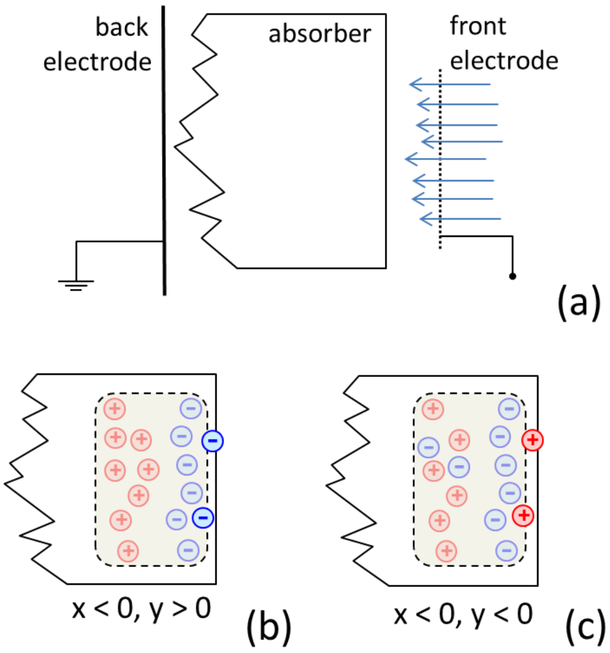

Figure S11. Schematic electrode configuration of an absorber with a grounded back contact and a transparent front electrode for SPV measurements (a) and schematics for preferential separation of photogenerated electrons towards the surface and preferential trapping of electrons or holes near the surface of the absorber ((b) and (c), respectively).

Table S1. Summarizing Energy Levels

Material	Band Gap, E_g (eV)	Flat Band Potential, φ_{fb} (V vs. RHE)	Work Function, Φ (eV vs. vacuum)	Valence Band Offset, $\lvert E_V - E_F \rvert$ (eV)
$CuBi_2O_4$	~1.6 [1]	~1.12 [1]	5.8 [1]	0.19 [1]
CdS	2.4-2.5 [6, 7]		4.1-4.3 [6] [this work]	2.5 [this work]
$BiVO_4$	2.5 [3, 8]	~0.37 [this work]		2.4 [this work]
Ga_2O_3	4.8 [9]		3.63 [this work]	4.6 [this work]
TiO_2	3.2 [10, 11]		4.49-4.60 [12, 13] [this work]	3.3 [this work]

References:

[1] A. Song, P. Plate, A. Chemseddine, F. Wang, F. F. Abdi, M. Wollgarten, R. van de Krol and S. P. Berglund, *J. Mater. Chem. A*, 2019, **7**, 9183-9194.

[2] F. Wang, W. Septina, A. Chemseddine, F. F. Abdi, D. Friedrich, P. Bogdanoff, R. van de Krol, S. D. Tilley and S. P. Berglund, *J. Am. Chem. Soc.*, 2017, **139**, 15094-15103.

[3] F. F. Abdi, L. Han, A. H. M. Smets, M. Zeman, B. Dam and R. van de Krol, *Nat. Commun.*, 2013, **4**, 2195.

[4] F. F. Abdi and R. van de Krol, *J. Phys. Chem. C*, 2012, **116**, 9398-9404.

[5] T. Dittrich, S. Fengler, *Surface Photovoltage Analysis of Photoactive Materials*, 2020.

[6] A. Cortes, H. Gomez, R. Marotti, G. Riveros and E. Dalchiele, *Sol. Energy Mater. Sol. cells,* 2004, **82**, 21-34.

[7] A. Song, P. Bogdanoff, A. Esau, I. Y. Ahmet, I. Levine, T. Dittrich, T. Unold, R. van de Krol and S. P. Berglund, *ACS Appl. Mater. Interfaces*, 2020, **12**, 13959-13970.

[8] D. K. Lee and K.-S. Choi, *Nat. Energy,* 2018, **3**, 53-60.

[9] M. Mohamed, K. Irmscher, C. Janowitz, Z. Galazka, R. Manzke and R. Fornari, *Appl. Phys. Lett.*, 2012, **101**, 132106.

[10] V. Stevanović, S. Lany, D. S. Ginley, W. Tumas and A. Zunger, *Phys. Chem. Chem. Phys.,* 2014, **16**, 3706-3714.

[11] Y. Xu and M. A. Schoonen, *Am. Mineral.*, 2000, **85**, 543-556.

[12] A. Orendorz, J. Wüsten, C. Ziegler and H. Gnaser, *Appl. Surf. Sci.,* 2005, **252**, 85-88.

[13] J. M. Bolts and M. S. Wrighton, *J. Phys. Chem.,* 1976, **80**, 2641-2645.
